# Reduction of PTEN protein and loss of *epidermal growth factor receptor* gene mutation in lung cancer with natural resistance to gefitinib (IRESSA)

**DOI:** 10.1038/sj.bjc.6602559

**Published:** 2005-05-03

**Authors:** Y Kokubo, A Gemma, R Noro, M Seike, K Kataoka, K Matsuda, T Okano, Y Minegishi, A Yoshimura, M Shibuya, S Kudoh

**Affiliations:** 1 Fourth Department of Internal Medicine, Nippon Medical School, 1-1-5 Sendagi, Bunkyo-ku, Tokyo 113-8602, Japan; 2 Division of Respiratory Medicine, Tokyo Metropolitan Cancer and Infection Center, Komagome Hospital, 3-18-22 Honkomagome, Bunkyo-ku, Tokyo 113-8677, Japan

**Keywords:** gefitinib, PTEN, Akt, *epidermal growth factor receptor(EGFR)* gene, mutation, natural resistance

## Abstract

Gefitinib (IRESSA), an epidermal growth factor receptor (EGFR) tyrosine kinase (TK) inhibitor, has antitumour activity in the advanced non-small-cell lung cancer (NSCLC) setting. However, in chemotherapy-naïve patients with advanced NSCLC, the addition of gefitinib to standard chemotherapy regimens failed to increase survival. These results suggest the need for improved patient selection and combination rationales for targeted therapies. We have identified subpopulations of an adenocarcinoma cell line that are naturally resistant to gefitinib, and have analysed the cDNA expression profiles, genomic status of *EGFR* gene and the effect of gefitinib on signalling pathways in these cell lines in order to identify key mechanisms for naturally acquired resistance to gefitinib. Gefitinib-resistant subpopulations demonstrated increased Akt phosphorylation (not inhibited by gefitinib), reduced PTEN protein expression and loss of the *EGFR* gene mutation when compared with parental cell lines. These differences in Akt and PTEN protein expression were not evident from the cDNA array profiles. These data suggests that (1) the *EGFR* gene mutation may be possibly lost in some cancer cells with other additional mechanisms for activating Akt, (2) reintroduction of PTEN or pharmacological downregulation of the constitutive PI3K–Akt-pathway activity may be an attractive therapeutic strategy in cancers with gefitinib resistance.

The epidermal growth factor receptor (EGFR) is a 170-kDa protein composed of an extracellular ligand-binding domain, a short transmembrane domain and an intracellular domain with intrinsic tyrosine kinase (TK) activity (Cohen *et al*, 1982; Carpenter and Cohen, 1990). High expression of EGFR has been reported in various epithelial malignant tumours, including lung cancer, and has been shown to play a causal role in disease progression (Ozanne *et al*, 1986; Haeder *et al*, 1988). Epidermal growth factor receptor is, therefore, a promising molecular therapeutic target in various tumour types, including lung cancer.

Gefitinib (IRESSA) (4-(3-chloro-4-fluoroanilino)-7-methoxy-6-(3-morpholinopropoxy)-quinazoline) is an orally active EGFR-TK inhibitor that inhibits EGFR signalling (Rusch *et al*, 1993; Salomon *et al*, 1995). Phase I trials of gefitinib in patients with solid tumours refractory to standard chemotherapy have shown good tolerability and evidence of antitumour activity (Ferry *et al*, 2000; Ranson *et al*, 2002). IDEAL (IRESSA Dose Evaluation in Advanced Lung cancer) 1 and 2 were randomised phase II trials in patients with non-small-cell lung cancer (NSCLC) refractory to platinum-based chemotherapy. These trials demonstrated that gefitinib was active and generally well tolerated: response rates were 18.4 and 11.8%, respectively, and the predominant toxicities were mild or moderate skin rash and diarrhoea (Fukuoka *et al*, 2003; Kris *et al*, 2003). The INTACT (IRESSA NSCLC Trial Assessing Combination Treatment) 1 and 2 phase III trials compared first-line chemotherapy with and without gefitinib in patients with advanced NSCLC and demonstrated similar survival outcomes in both treatment arms (Giaccone *et al*, 2004; Herbst *et al*, 2004). These data indicate that improved patient selection and combination strategies are required for optimal utility of this targeted therapy.

Gefitinib exerts antitumour activity through inhibition of EGFR-TK, but its antitumour activity is not significantly correlated with tumour cell EGFR expression (Bailey *et al*, 2003). Recently, differences in the frequency of activating mutations in the *EGFR* gene between gefitinib responders and nonresponders were reported and these *EGFR* mutations seem to be predictive markers for sensitivity to gefitinib (Lynch *et al*, 2004; Paez *et al*, 2004). We examined sensitivity to gefitinib in NSCLC cell lines using the MTT (3-(4,5-dimethylthiazol-2-yl)-2,5-tetrazolium bromide) cell proliferation assay and identified a parent adenocarcinoma cell line, PC9, that was sensitive to gefitinib and two subpopulations, PC9/f9 and PC9/f14, that were resistant. These subpopulations of PC9 were established by artificial metastasis methods and resistance to gefitinib was therefore acquired naturally, without exposure to gefitinib. In order to identify additional key molecules involved in gefitinib resistance, we analysed expression profiles using cDNA array and genomic status of the *EGFR* gene in the parent cell line and subpopulations, and examined the effect of gefitinib on the downstream mediators of EGF-mediated signalling PI3K–Akt and Ras/MEK/Erk pathways (Olayioye *et al*, 2000).

## MATERIALS AND METHODS

### Cell lines

Using the MTT assay, we analysed the growth-inhibitory effect of gefitinib on nine NSCLC cell lines: A549, PC3, PC7, PC9, PC9/f9, PC9/f14 and PC14 adenocarcinoma cell lines, Lu65 large-cell carcinoma cell line and LK-2 squamous-cell carcinoma cell line. PC9/f9 (Takenaka *et al*, 2000) and PC9/f14 (Gemma *et al*, 2001) are highly metastatic sublines of PC9 established at Nippon Medical School using artificial metastasis methods. In an attempt to elucidate potential mechanisms of resistance to gefitinib, we evaluated cDNA expression profiles and their relationship to gefitinib resistance. We also specifically examined the effect of gefitinib on the PI3K–Akt and Ras/MEK/Erk pathways in PC9, PC9/f9 and PC9/f14 cells.

### Drugs and growth-inhibition assay

Gefitinib was provided by AstraZeneca, Macclesfield, UK and dissolved in dimethyl sulphoxide (DMSO) for *in vitro* studies. We used the colourimetric MTT assay (tetrazolium dye assay) to examine the activity of gefitinib on NSCLC cell lines (Mosmann, 1983). Cell suspensions (200 *μ*l 1 × 10^5^ cells ml^−1^) were seeded into the wells of a 96-well microtitre plate and 10 *μ*l of drug solution (various concentrations) was added. After incubation for 72 h at 37°C, 20 *μ*l of MTT solution (5 mg ml^−1^ in phosphate-buffered saline) was added to each well and incubated for a further 4 h at 37°C. After centrifugation at 1500 r.p.m. for 5 min, the medium was aspirated from each well and 200 *μ*l of DMSO was added to dissolve the formazan. The IC_50_ value was defined as the concentration needed for a 50% reduction in absorbance (560 nm) based on survival curves.

### RNA isolation, cDNA array hybridisation and analysis of hybridisation signals

Total RNA was isolated from each cell line using standard protocols described previously (Gemma *et al*, 1996, 1998). Messenger RNA was then purified from total RNA by incubation with oligo-dT-magnetic beads (Toyobo Co., Osaka, Japan). The ElectorGene Array System (GeneticLab Co. Ltd, Sapporo, Japan) was used for filter-based cDNA array analysis, as previously reported (Gemma *et al*, 2001). In this analysis, 1300 species of human DNA fragments, including cancer-related and drug-resistance-associated genes, as well as housekeeping and non-mammalian genes as negative controls, are spotted in duplicate on a filter. The probes were prepared by reverse transcription (Reverse Transcriptase, ReverTraAce (Toyobo Co., Osaka, Japan)), with a random 9 mer (Toyobo Co., Osaka, Japan) and 5 mg of poly A RNA. The probes were labelled with biotin by incorporation of biotin-16-deoxyuracil triphosphate during the synthesis of cDNA. The filters were preincubated in 20 ml of PerfectHyb (Toyobo Co., Osaka, Japan) at 68°C for 30 min. The biotin-labelled probes were denatured and added to the prehybridisation solution and the filters were incubated overnight at 68°C in the hybridisation mixture. After washing, specific signals on the filters were detected by the Imaging High-Chemilumi-Detection kit (Toyobo Co., Osaka, Japan). CDP-Star substrate (Tropix, Bedford, MA, USA) was used as the chemiluminescence substrate, and a chemiluminescence image of the filter was acquired by Fluor-S (Bio-Rad, Hercules, CA, USA). Gene-expression images were quantified by measuring the intensity of the signals using Imagene (BioDiscovery, Los Angeles, CA, USA) and filter signal intensity was analysed by the ElectorGene Finding System (GeneticLab Co. Ltd, Sapporo, Japan). The background threshold was set at a level three-fold higher than the negative control. Signal intensities were normalised by comparison with the expression of housekeeping genes, GAPDH (glyceraldehyde-3-phosphate dehydrogenase) and beta-actin. A three-fold difference in gene expression among the subpopulations and the parent cell line was considered to be significant, on the basis of the company's recommendation (Toyobo Co., Osaka, Japan).

### Effect of gefitinib on PI3K–Akt and Ras/MEK/Erk pathways in PC9, PC9/f9 and PC9/f14 cells

#### Exposure to gefitinib

PC9, PC9/f9 and PC9/f14 cells were serum-starved and treated with various concentrations of gefitinib (0, 5, 50, 500 and 5000 ng ml^−1^) for 2 h before exposure to 10 ng ml^−1^ EGF (BA-53) (Santa Cruz Biotechnology, Santa Cruz, CA, USA) for 5 min.

#### Western blot analysis

Lysis buffer containing 1% Triton X and 1% NP40 was added to the cells before sonication in a POLYTRON homogenizer (KINEMATICA, Littau-Lucerne, Switzerland). The lysates were cleared by centrifugation at 14 000 r.p.m. for 20 min, and then 10 *μ*l of lysate containing 10 *μ*g of protein were separated by SDS–polyacrylamide gel electrophoresis (PAGE). After PAGE, the proteins were transferred to nitrocellulose membranes and blotted with the following primary antibodies: PTEN A2B1 (Santa Cruz Biotechnology, Santa Cruz, CA, USA), and Akt, phospho-Akt (Ser473), p38 MAP kinase and phospho-p38 MAP kinase (Thr180/Tyr182) (all Cell Signaling Technology, Beverly, MA, USA). The membranes were then incubated with peroxidase-conjugated secondary antibodies and protein was detected with the ECL Western blotting detection reagents (Amersham, Buckinghamshire, UK) (Naishiro *et al*, 2001) and film-processor FPM100 film (Fuji Photo Film). These images were quantified by measuring the intensity of the signals using NIH Image (ImageJ1.32j).

### Genomic DNA analysis of the *PTEN* gene by polymerase chain reaction

From each genomic DNA sample, all exons of the *PTEN* gene were amplified separately with the polymerase chain reaction (PCR) primers previously described (Hosoya *et al*, 1999) using the Gene Amp XL PCR kit (Perkin Elmer/Roche, Branchburg, NJ, USA). Polymerase chain reaction conditions for genomic DNA analysis were as follows: 40 cycles at 94°C for 40 s, at 60°C for 30 s and at 68°C for 90 s, followed by 68°C for 8 min. Each reaction mixture contained 1 × XL buffer, 200 *μ*M deoxynucleotide triphosphate, 1100 *μ*M Mg(OAc)2, 0.5 U rTth DNA polymerase XL, 0.3 mM of each primer (one of each pair) (Hosoya *et al*, 1999) and 25 ng of genomic DNA. Polymerase chain reaction products were loaded on 1.2% agarose gels. After electrophoresis, the gels were analysed.

### Mutation analysis of the *EGFR* gene

Polymerase chain reaction–single strand conformation polymorphism (PCR–SSCP) analysis was performed as previously described (Gemma A, *et al*, 1996, 1998). Each of the three exons (18, 19 and 21) of the *EGFR* gene was amplified separately using reported PCR primers (Lynch *et al*, 2004). Polymerase chain reaction was performed using the Gene AMP XL PCR kit (Perkin–Elmer Corp./Roche, Branchburg, NJ, USA) as described above. The PCR reaction mixture contained XL buffer containing 1100 *μ*M Mg(OAc)_2_, 200 *μ*M deoxynucleotide triphosphate, 0.1 mM of each primer labelled with 5-IAF (Amersham Pharmacia Biotech, Uppsala, Sweden), 0.5 U of rTth DNA polymerase and 25 ng of genomic DNA. The 5-IAF-labelled PCR products were denatured, cooled on ice, and loaded on neutral 6% polyacrylamide gels with or without 5% (vol vol^−1^) glycerol. Following electrophoresis, the gels were analysed using the FluorImager 595 (Amersham Pharmacia Biotech, Uppsala, Sweden). DNA sequence analysis was performed as previously described (Gemma A, *et al*, 1996, 1998). Aberrant bands were cut from the gel and further amplified by PCR using sequencing primers with the M13 sequence (TGTAAAACGACGGCCAGT) added to the appropriate PCR primers. Sequencing reaction was performed, and the products were purified and sequenced using a fluorescent automated sequencer (Perkin–Elmer Corp./Applied Biosystem, Inc., Foster City CA, USA).

### Multicolour fluorescent *in situ* hybridisation (FISH) analyses of metaphase preparations from cancer cell line subpopulation

Multicolour-FISH on metaphase preparations was performed using Spectra Vysion probes according to the instructions of the manufacturer (Vysis, Downers Grove, IL, USA). Images were visualised by an epifluorescence microscope (Zeiss, Oberhochen, Germany) and analysed using an Applied Imaging CytoVision Work station (Newcastle, UK, USA). A total of 20 metaphase cells were analysed in each subpopulation.

## RESULTS

### Effect of gefitinib on cell growth *in vitro*

The IC_50_ values of gefitinib on nine NSCLC cell lines, as determined by the MTT assay, are summarised in Table 1. In accordance with the minimal steady-state concentration reported in the clinical trial (264 ng ml^−1^; 0.59 *μ*M), PC9 and A549 seemed to be sensitive to gefitinib. Interestingly, two subpopulations of the PC9 cell line, PC9/f9 and PC9/f14, showed resistance to gefitinib even though the parent cell line was sensitive. A highly metastatic human lung adenocarcinoma cell line, PC9/f9, was established in an experimental metastasis model by repeated inoculation of PC9 cells in nude mice and subsequent culture of the tumour cells harvested from pulmonary metastatic foci (Takenaka *et al*, 2000). PC9/f14 was established by five additional inoculations (Gemma *et al*, 2001). In the matrigel invasion assay, the PC9/f9 and PC9/f14 cells showed higher invasive activity than the parent PC9 cells (data not shown). The resistance to gefitinib in PC9/f9 and PC9/f14 cells was therefore acquired naturally in the absence of exposure to gefitinib.

### cDNA array analysis in PC9, PC9/f9 and PC9/f14 cells

**Figure 1:**
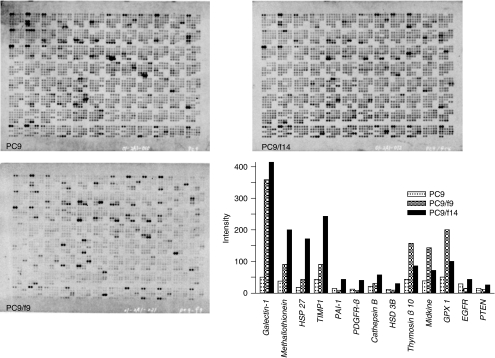
Expression profiles of the sensitive cell line PC9 and resistant subpopulations PC9/f9 and PC9/f14 using cDNA array.

In order to identify potential key molecules for natural resistance to gefitinib, we analysed the expression profiles of the PC9, PC9/f9 and PC9/f14 cell lines using cDNA array, and examined the effect of gefitinib on the EGF downstream mediators PI3K–Akt and Ras/MEK/Erk. Significant enhancement of four and eight genes was identified in the PC9/f9 and PC9/f14 cell lines, respectively, compared with the parent cell line. Only *Galectin-1* was overexpressed in both of the resistant cell lines and *lactate dehydrogenase A* was downregulated. There were no significant differences in *EGFR* expression, nor were *Ras*, *PTEN*, *PI3K*, *Akt*, *Raf* or *ERK1/2* differentially expressed (Figure 1).

### Phosphorylation of Akt in PC9, PC9/f9 and PC9/f14 cells

**Figure 2:**
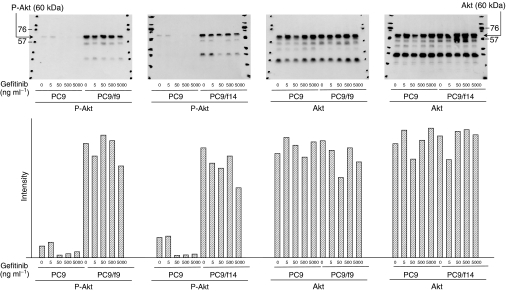
Expression and phosphorylation state of Akt in the sensitive cell line PC9 and resistant subpopulations PC9/f9 and PC9/f14, and dose-dependent effect of gefitinib.

We examined expression and phosphorylation (Ser473) of Akt in PC9, PC9/f9 and PC9/f14 cells using Western blot analysis. There were no significant differences in Akt expression between the parent cell line and subpopulations. However, PC9/f9 and PC9/f14 cells demonstrated increased Akt phosphorylation compared with PC9 cells (Figure 2).

### Expression of PTEN in PC9, PC9/f9 and PC9/f14 cells

**Figure 3:**
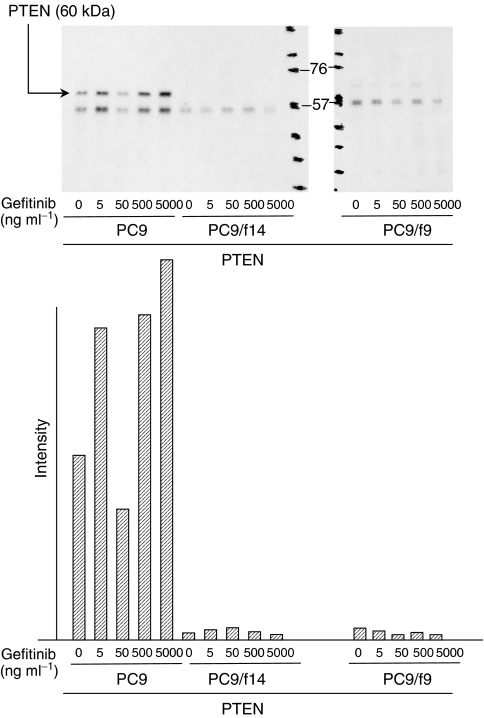
Expression of PTEN in the sensitive cell line PC9 and resistant subpopulations PC9/f9 and PC9/f14, and dose-dependent effect of gefitinib.

**Figure 4:**
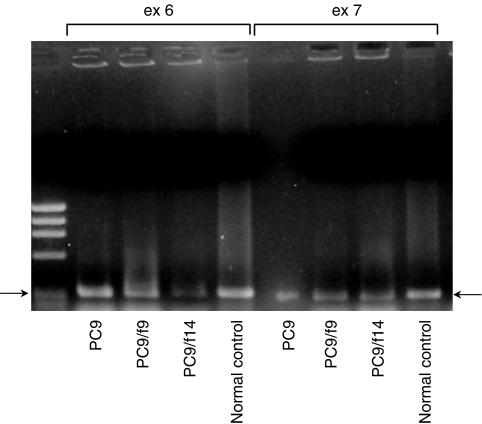
Genomic DNA analysis of the *PTEN* gene in sensitive cell line PC9 and resistant subpopulations PC9/f9 and PC9/f14.

We also examined expression of PTEN, a phosphatase that can dephosphorylyse position D3 of phosphatidylinositol-3,4,5 triphosphatase and which is a major negative regulator of the PI3 kinase/Akt signalling pathway (Cantley and Neel, 1999; Simpson and Parsons, 2001). PC9 demonstrated moderate expression of PTEN and there was minimal or absent expression of PTEN in PC9/f9 and PC9/f14 cells (Figure 3). Frequent homozygous deletion of the *PTEN* gene has been reported in lung cancer (Kohno *et al*, 1998; Ali *et al*, 1999; Vivanco and Sawyers, 2002), so we performed screening of genomic DNA analysis by PCR. We detected no homozygous deletions of the *PTEN* gene in any of the three subpopulations of the cell line examined (Figure 4).

### Expression and phosphorylation state of p38 MAP kinase in PC9, PC9/f9 and PC9/f14 cells

**Figure 5:**
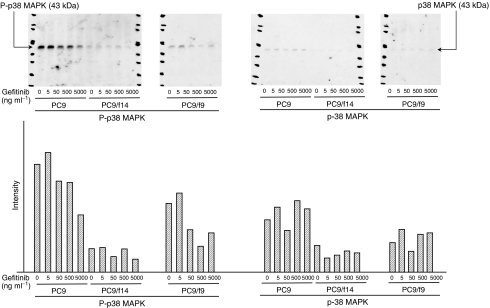
Expression and phosphorylation state of p38 MAP kinase in the sensitive cell line PC9 and resistant subpopulations PC9/f9 and PC9/f14, and dose-dependent effect of gefitinib.

We then examined the expression and phosphorylation state of p38 MAP kinase in PC9, PC9/f9 and PC9/f14 cells. p38 MAP kinase is activated by a variety of cellular stresses including osmotic shock, inflammatory cytokines, ultraviolet light, and growth factors. Phospho-p38 MAP kinase antibody detects p38 MAP kinase only when activated by dual phosphorylation at Thr180 and Tyr182. PC9 demonstrated activated p38 but only minimally activated p38 was observed in PC9/f9 and PC9/f14 cells (Figure 5).

### Mutation of *EGFR* gene in these cell lines

**Figure 6:**
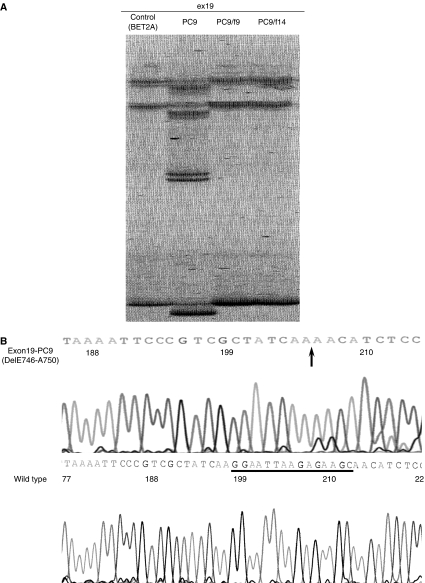
(**A**) Polymerase chain reaction–SSCP analysis of the *EGFR* gene in PC9, PC9/f9 and PC9/f14. Aberrant bands were shown only in PC9. (**B**) Sequence of aberrant and normal bands. These sequence analyses showed deletion of the *EGFR* gene in PC9.

**Figure 7:**
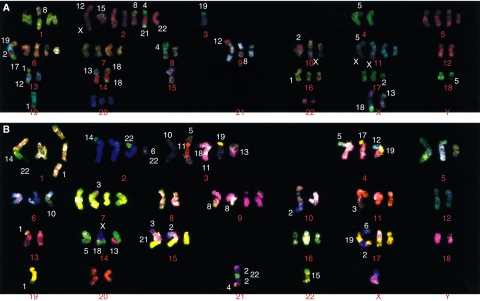
Multicolour FISH analyses of PC9 (**A**) and PC9/f14 (**B**). Chromosome numbers of PC9 and PC9/f14 are 61 and 60, respectively. Common aberrant chromosomes are eight.

Polymerase chain reaction–SSCP analysis was performed on these three lung cancer cell line subpopulations. The reported mutation in the *EGFR* gene was detected in parent cell line, PC9 (Arao T, *et al*, 2004), but no mutation were detected in PC9/f9 and PC9/f14 (Figure 6). We performed karyotype analyses using multicolour FISH analysis to confirm that these subpopulations have same origin. The multicolour FISH analyses demonstrated that these subpopulations had similar chromosome numbers and chromosomal rearrangements (at least, eight common rearrangements) (Figure 7). These results showed that these subpopulations with naturally acquired resistance to gefitinib lost the *EGFR* gene mutation.

### Effect of gefitinib on phosphorylation of Akt and p38 MAP kinase and expression of PTEN

Using Western blot analysis, we also examined the effect of gefitinib on PTEN expression, and expression and phosphorylation of Akt and p38 MAP kinase in PC9, PC9/f9 and PC9/f14 cells. While gefitinib had no influence on Akt phosphorylation in PC9/f9 and PC9/f14 cells, it inhibited Akt phosphorylation in PC9 cells, with complete inhibition of Akt phosphorylation observed at gefitinib concentrations ⩾0.1 *μ*M (44.7 ng ml^−1^). Notably, this concentration is substantially lower than that reported as the minimal steady-state concentration in clinical trials (Ranson *et al*, 2002). In PC9 cells, gefitinib was associated with increased PTEN expression, but there was absent or only minimal expression of PTEN in PC9/f9 and PC9/f14 cells and gefitinib did not influence expression. Gefitinib did not influence the expression or phosphorylation state of p38 MAP kinase (Figures 2, 3 and 5).

## DISCUSSION

Gefitinib exerts antitumour activity through inhibition of EGFR-TK (Rusch *et al*, 1993; Salomon *et al*, 1995), but its antitumour activity cannot be predicted by EGFR expression in tumours (Bailey *et al*, 2003). Recent reports showing differences in the frequency of activating mutations in the *EGFR* gene between gefitinib responders and nonresponders suggest that these *EGFR* mutations are predictive markers for sensitivity to gefitinib (Lynch *et al*, 2004; Paez *et al*, 2004). However, there are also sensitive tumors to gefitinib without the *EGFR* gene mutation. We examined the sensitivity of various NSCLC cell lines to gefitinib using the MTT assay and attempted to identify potential key molecules involved in gefitinib resistance. We identified PC9, a parent adenocarcinoma cell line sensitive to gefitinib, and two resistant subpopulations, PC9/f9 and PC9/f14. As the artificial metastasis method was used for subpopulation generation, PC9/f9 and PC9/f14 had highly metastatic potential and natural resistance to gefitinib.

We analysed the expression profiles using cDNA array, genomic status of the *EGFR* gene, and examined the effect of gefitinib on the downstream mediators of EGF-mediated signalling PI3K–Akt and Ras/MEK/Erk (Olayioye *et al*, 2000) pathways in these subpopulations. Both resistant subpopulations demonstrated increased activation of Akt, reduced expression of PTEN and loss of the *EGFR* gene mutation, compared with the parent cell line. Gefitinib inhibited Akt phosphorylation in the gefitinib-sensitive parent cell line but not in the resistant subpopulations PC9/f9 and PC9/f14.

She *et al* (2003) reported that the MDA468 breast cancer cell line, which lacks PTEN function, was resistant to gefitinib. Reconstitution of PTEN function in MDA468 cells through tet-inducible expression restored gefitinib sensitivity and re-established EGFR-stimulated Akt signalling. Bianco *et al* (2003) also reported that loss of PTEN in MDA468 counteracted the antitumour action of EGFR-TK inhibitors and that restoration of PTEN by retroviral infection was associated with gefitinib-mediated inhibition of Akt phosphorylation, and increased apoptosis and cell-cycle delay.

In accordance with these data, our results demonstrated loss of PTEN protein and overactivation of Akt in NSCLC subpopulations with highly metastatic potential and natural resistance to gefitinib, and indicated that PTEN protein level and Akt phosphorylation state may be additional key predictors of *de novo* resistance to gefitinib. Reintroduction of PTEN or pharmacological downregulation of constitutive PI3K–Akt-pathway activity might potentially sensitise lung carcinomas to gefitinib, although further preclinical studies are required.

There are many reports of Akt-promoted tumour invasion and metastasis. Park *et al* (2001) showed that Akt1 induced extracellular matrix invasion and matrix metalloproteinase (MMP)-2 activity in mouse mammary epithelial cells. Kim *et al* (2001) also demonstrated that Akt/protein kinase-B promoted cancer cell invasion via increased motility and MMP production. Overexpression of Akt2 was also reported to lead to upregulation of beta-1 integrins and increased invasion and metastasis of human breast and ovarian cancer cells by Arboleda *et al* (2003). We established highly metastatic human lung adenocarcinoma cell subpopulations, PC9/f9 and PC9/f14, in an experimental metastasis model by repeated inoculation of PC9 cells in nude mice and subsequent culture of the tumour cells that were harvested from pulmonary metastatic foci. The PC9/f9 and PC9/f14 cells showed higher invasive activity in the matrigel invasion assay than the parent PC9 cells (data not shown). Our data indicated that loss of PTEN and overactivation of Akt during the repeated process of artificial metastasis were associated with increased invasive activity and may have contributed to natural resistance to gefitinib. Loss of the *EGFR* gene mutation in PC9/f9 and PC9/f14 indicated that constitutive Akt phosphorylation by *EGFR* gene mutation does not seem to be necessary in some cancer cells with other additional mechanisms of activating Akt.

In the expression profile analysis using cDNA array, we could identify several molecules that were significantly up- or downregulated in the gefitinib-resistant subpopulations compared with the parent cell line. However, while the resistant subpopulations demonstrated increased activation of Akt and reduced expression of PTEN in Western blot analysis, we could not identify significant differences in PTEN and *Akt* using cDNA array. Furthermore, Western blot analysis failed to demonstrate a difference in Akt gene expression between parent and resistant subpopulations. The failure to demonstrate homozygous deletions of the *PTEN* gene in these subpopulations and differences in *PTEN* mRNA levels between the sensitive and resistant cell subpopulations indicates that the reduced PTEN protein expression in these gefitinib-resistant cells may be the result of post-translational regulation or partial genomic alterations. Our data indicate that, at this time, gene expression profile analysis may not be sufficient for the identification of potential key molecules in gefitinib resistance, and protein expression and evaluation of phosphorylation status may be more informative. The reduced phosphorylation of p38 observed in PC9/f9 and PC9/f14 cells in our study indicates that gefitinib resistance may be affected through a negative feedback mechanism.

Homozygous deletion of the *PTEN* gene has been reported in lung cancer (Kohno *et al*, 1998; Ali *et al*, 1999; Vivanco and Sawyers, 2002). The results of our study indicate that PTEN protein expression and Akt phosphorylation may be candidate markers of gefitinib sensitivity; conversely, lack of PTEN expression and Akt phosphorylation may be candidate markers of gefitinib resistance. Those tumours with activating mutations in the *EGFR* gene but which lack PTEN may be resistant to gefitinib, as PTEN is located downstream of the EGFR. Since Akt phosphorylation is influenced by activating mutations in the *EGFR* gene and loss of PTEN function, Akt phosphorylation alone could not be a secondary marker of these *EGFR* mutations. More extensive clinical study using biopsy samples may be useful for further evaluation of PTEN protein expression as a marker for gefitinib sensitivity.

Finally, the *EGFR* gene mutation may be lost in some cancer cells with other additional mechanisms of activating Akt. This alteration might be a novel mechanism and/or marker for acquired resistance to gefitinib. Reintroduction of PTEN or pharmacological downregulation of the constitutive PI3K–Akt-pathway activity may be an attractive therapeutic strategy in cancers without the *EGFR* gene mutation. The existence of these cell line subpopulations will facilitate investigation of EGF-mediated signalling PI3K–Akt in human cancers.

## Figures and Tables

**Table 1:** *In vitro* growth-inhibitory activity of gefitinib on NSCLC cell lines in the MTT assay

**Cell line**	**Cell type**	**IC_50_ (*μ*M)**
PC9	Adenocarcinoma	2.0 × 10^−3^
PC9/f9	Highly metastatic subpopulation of PC9	>20
PC9/f14	Highly metastatic subpopulation of PC9	19
A549	Adenocarcinoma	1.8 × 10^−2^
PC7	Adenocarcinoma	>20
PC14	Adenocarcinoma	8.0
PC3	Adenocarcinoma	>20
LU65	Large-cell carcinoma	13
LK-2	Squamous-cell carcinoma	20
